# Supplementary figures and images for: OrthoSNAP: A tree splitting and pruning algorithm for retrieving single-copy orthologs from gene family trees
Source: PLoS Biol. 2022 Oct 13;20(10):e3001827. doi: 10.1371/journal.pbio.3001827 (PMC9595520; doi:10.1371/journal.pbio.3001827)

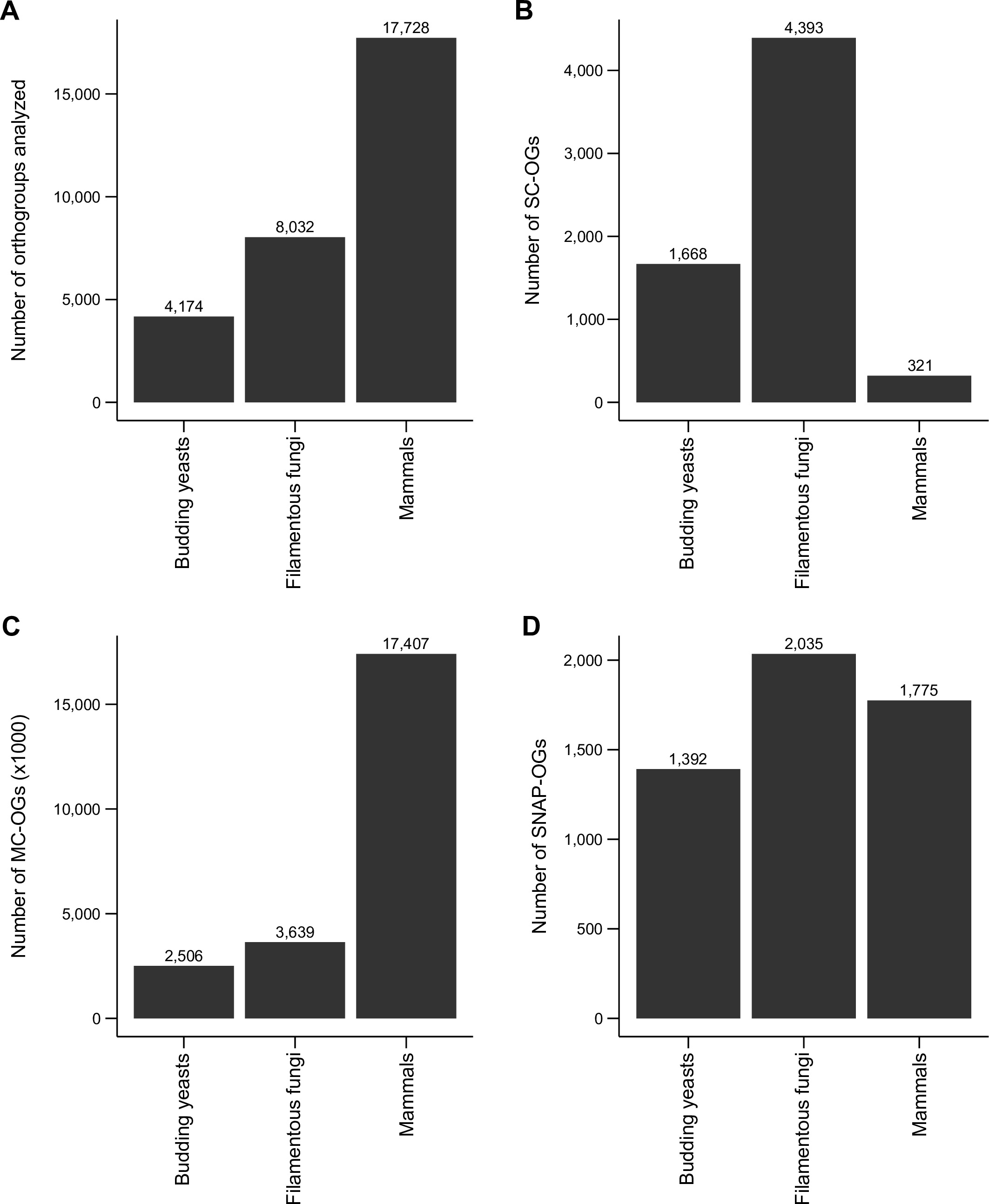

Supplement: S1 Fig — (A) The total number of orthogroups with at least 50% ortholog occupancy for each dataset. (B) The number of single-copy orthologs (SC-OGs) for each dataset (with at least 50% taxon occupancy). (C) The number of multicopy orthologs (or orthologous groups of genes wherein 1 or more species is represented by 2 or more sequences; MC-OGs) for each dataset (with at least 50% taxon occupancy). (D) The number of SNAP-OGs identified in each dataset (with at least 50% taxon occupancy). Note that the numbers depicted in panel A reflect the sum of the numbers of SC-OGs and MC-OGs in panels B and C. The data underlying this figure can be found in figshare (doi: 10.6084/m9.figshare.16875904). (TIF) [file pbio.3001827.s001.tif]

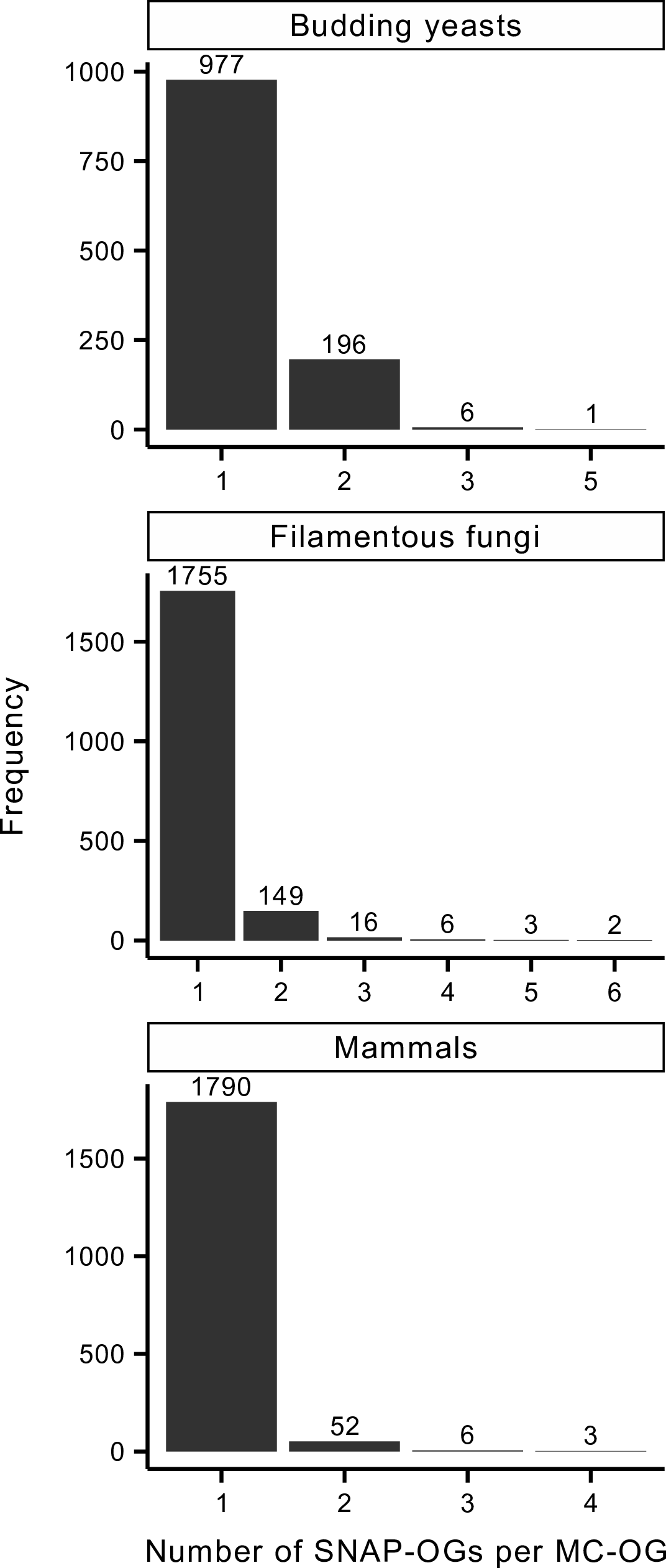

Supplement: S2 Fig — The number of SNAP-OGs per orthologous group of genes is depicted on the x-axis. For example, in the budding yeasts dataset, 977 gene families had 1 SNAP-OG each. The highest number of SNAP-OGs identified in a single orthologous group of genes in each dataset were as follows: in budding yeasts, 5 SNAP-OGs were identified in 1 orthologous group of genes that encode transcriptional activators; in filamentous fungi, 5 SNAP-OGs were identified in each of 2 orthologous groups of genes that encode multifacilitator superfamily transporters and amino acid permeases; and in mammals, 4 SNAP-OGs were identified in each of 3 orthologous groups of genes that encode voltage-gated potassium channels, casein kinases, and a tropomyosin family of actin-binding proteins. The data underlying this figure can be found in figshare (doi: 10.6084/m9.figshare.16875904). (TIF) [file pbio.3001827.s002.tif]

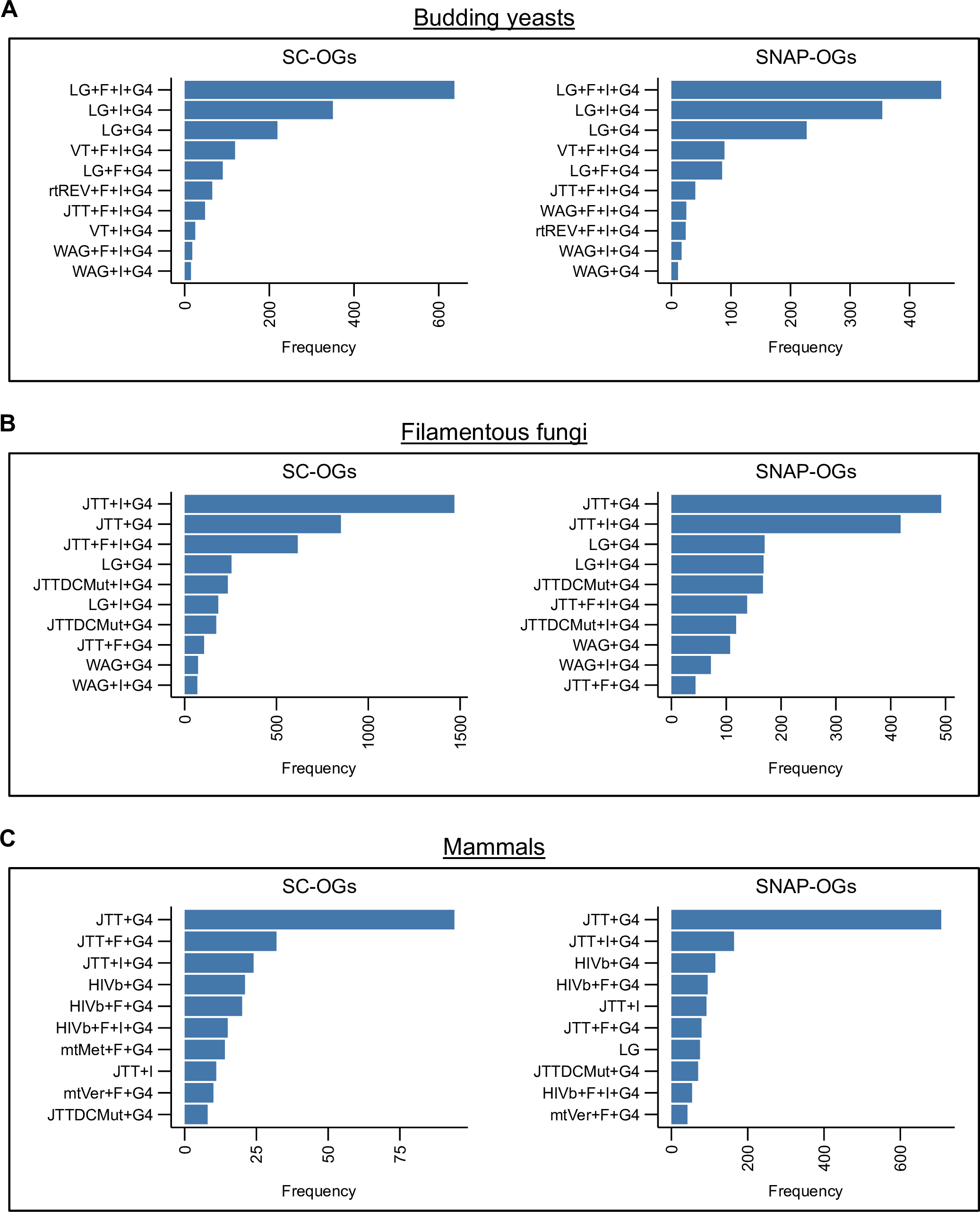

Supplement: S3 Fig — The top 10 most frequently observed best-fitting substitutions models were similar between SC-OGs and SNAP-OGs among (A) 1,668 SC-OGs and 1,392 SNAP-OGs in budding yeasts, (B) 4,393 SC-OGs and 2,035 SNAP-OGs in filamentous fungi, and (C) 321 SC-OGs and 1,775 SNAP-OGs in mammals. For example, the LG+F+I+G4 model was the most frequently observed best-fitting substitution model in SC-OGs and SNAP-OGs from budding yeasts. The data underlying this figure can be found in figshare (doi: 10.6084/m9.figshare.16875904). (TIF) [file pbio.3001827.s003.tif]

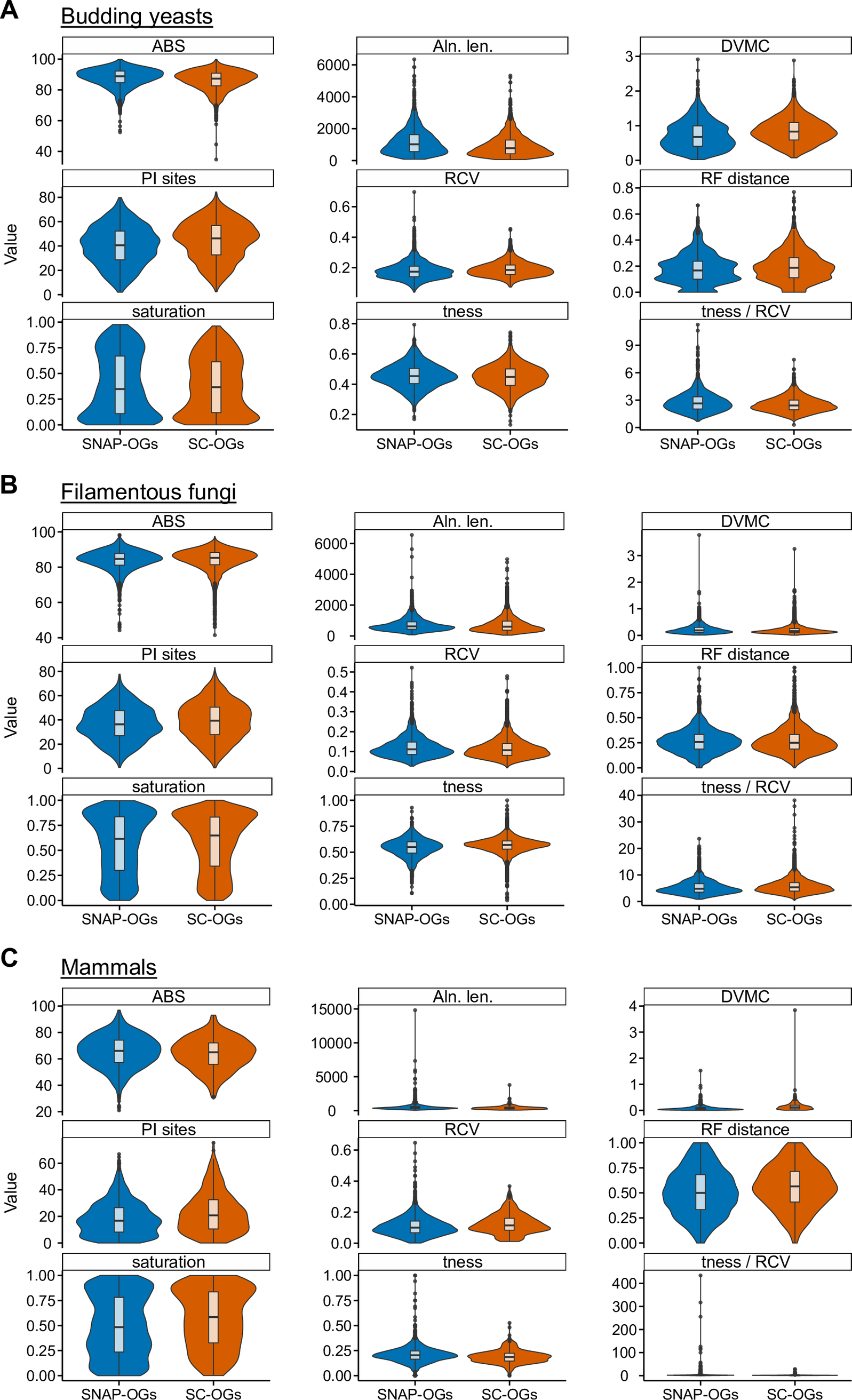

Supplement: S4 Fig — Boxplot and violin plot distributions of 9 properties representative of phylogenetic information are depicted SNAP-OGs (blue) and SC-OGs (orange) in the (A) 1,668 SC-OGs and 1,392 SNAP-OGs in budding yeasts, (B) 4,393 SC-OGs and 2,035 SNAP-OGs in filamentous fungi, and (C) 321 SC-OGs and 1,775 SNAP-OGs in mammals. Abbreviations are as follows: average bootstrap support (ABS), degree of violation of the molecular clock (DVMC), relative composition variability, Robinson-Foulds distance (RF distance), alignment length (Aln. len.), the number of parsimony informative sites (PI sites), saturation, treeness (tness), and treeness/RCV (tness/RCV). The data underlying this figure can be found in figshare (doi: 10.6084/m9.figshare.16875904). (TIF) [file pbio.3001827.s004.tif]

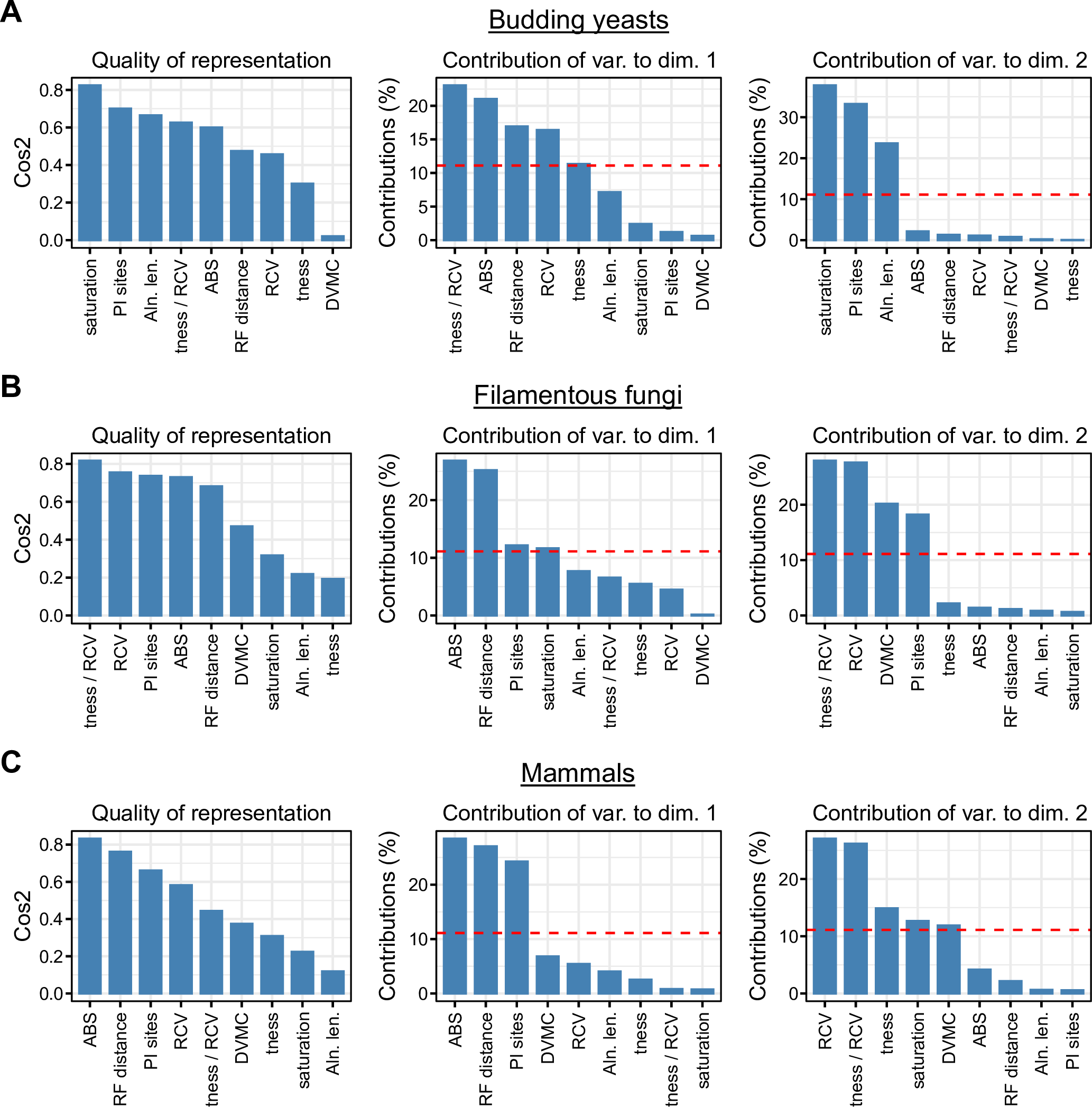

Supplement: S5 Fig — Principal component analysis was used to qualitatively compare the similarities and differences between SNAP-OGs and SC-OGs (Fig 3). The leftmost figure in each panel of budding yeasts (A), filamentous fungi (B), and mammals (C) represents the quality of representation for each property across all principal components. The next 2 figures depict the contribution of each property (or variable) to the first and second dimension in reduced dimensional space. The red dashed line represents equal contributions from each variable. The data underlying this figure can be found in figshare (doi: 10.6084/m9.figshare.16875904). (TIF) [file pbio.3001827.s005.tif]

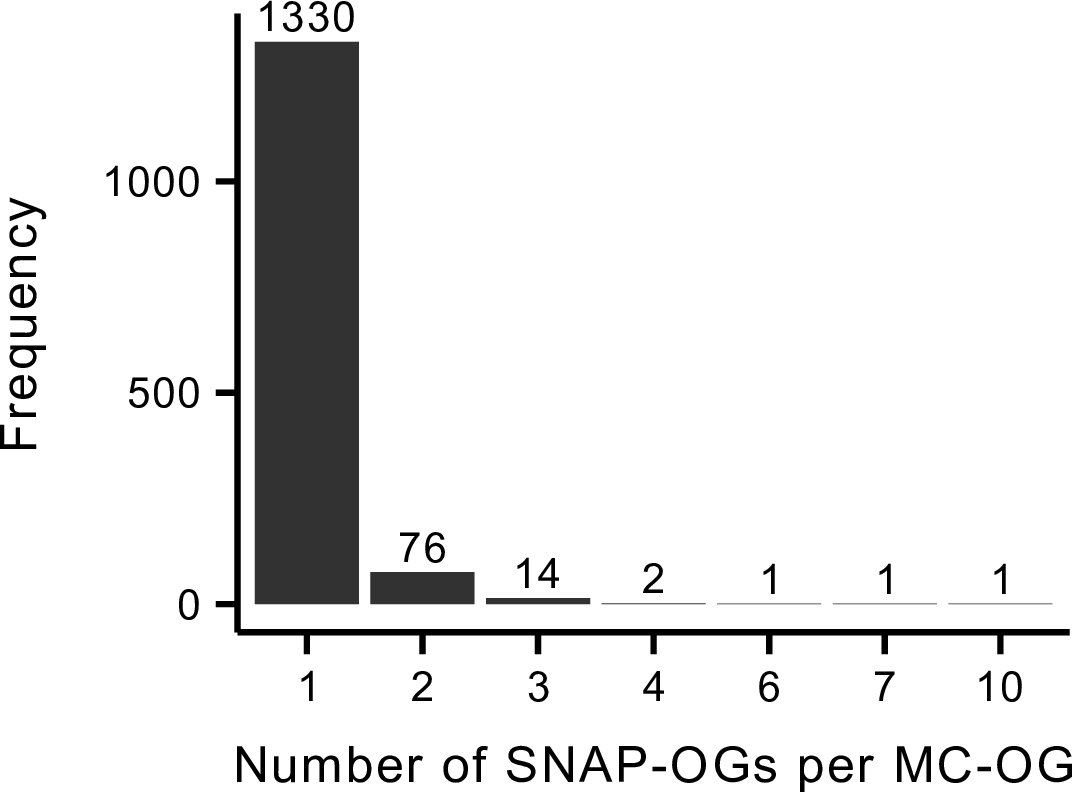

Supplement: S6 Fig — The number of SNAP-OGs per orthologous group of genes is depicted on the x-axis. For example, a single SNAP-OG was identified in 1,330 gene families with 2 or more homologs in 1 or more species, whereas 4 SNAP-OGs were identified in 2 gene families with 2 or more homologs in 1 or more species. The data underlying this figure can be found in figshare (doi: 10.6084/m9.figshare.16875904). (TIF) [file pbio.3001827.s006.tif]

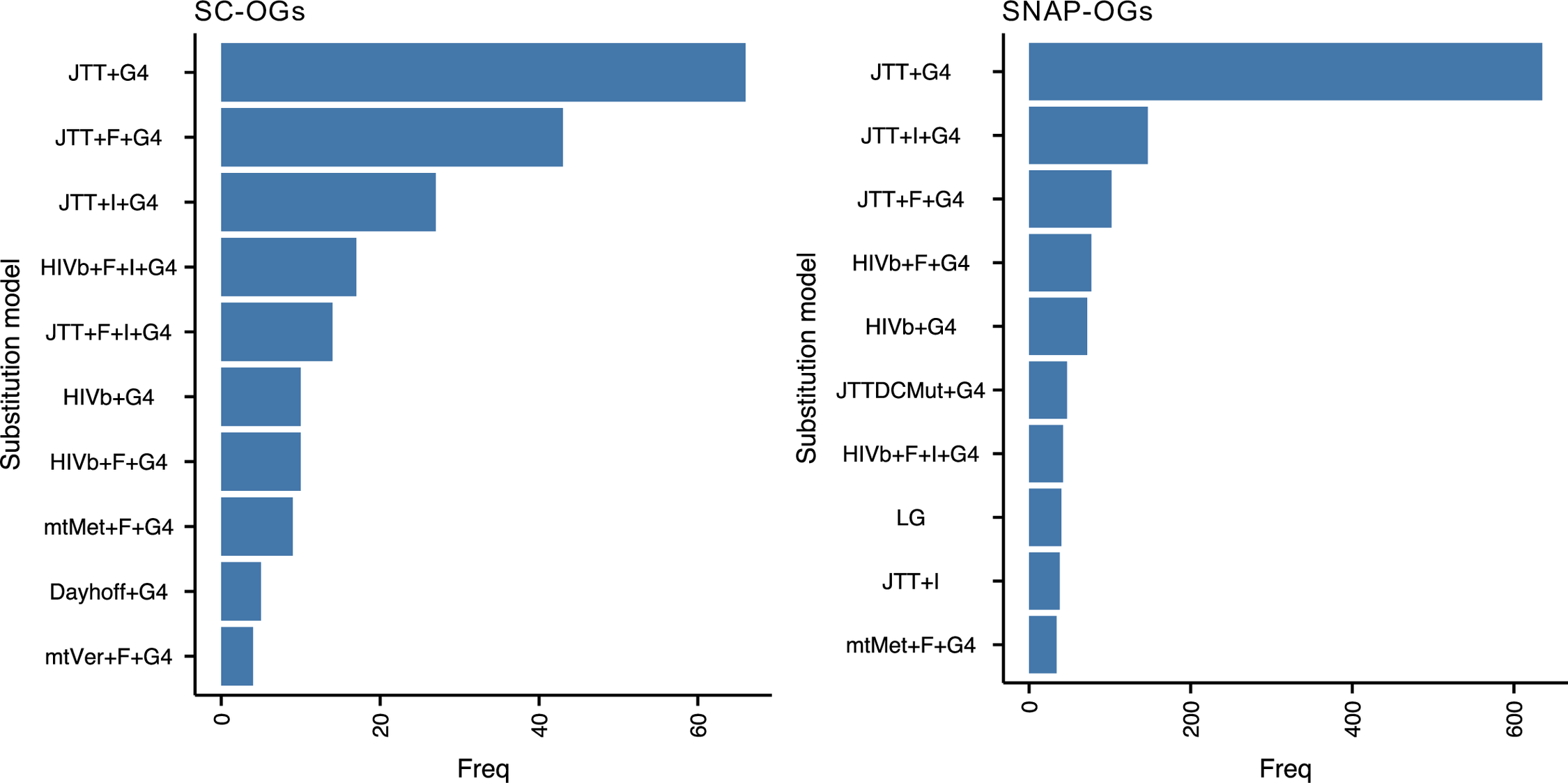

Supplement: S7 Fig — Similar best-fitting substitutions models were observed between 252 SC-OGs and 1,428 SNAP-OGs in a dataset of mammals, which was used to investigate patterns of support in a contentious branch in the tree of life concerning deep evolutionary relationships among placental mammals. The data underlying this figure can be found in figshare (doi: 10.6084/m9.figshare.16875904). (TIF) [file pbio.3001827.s007.tif]

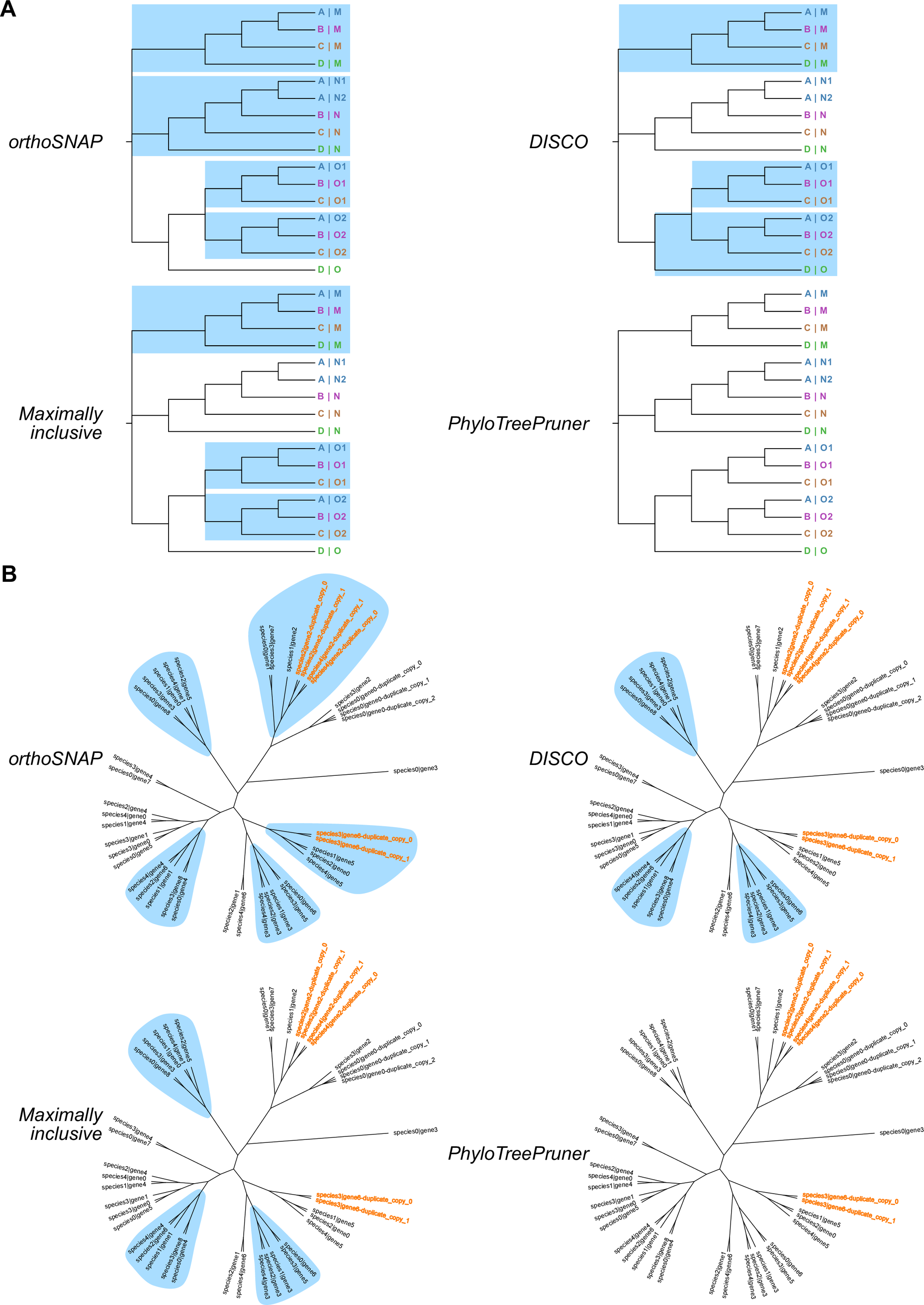

Supplement: S8 Fig — Using the phylogeny presented in Fig 1B (panel A) and Fig 2B (panel B), different tree decomposition algorithms are compared. (A) OrthoSNAP will identify 4 SNAP-OGs, whereas DISCO and the maximally inclusive strategies will each identify 3 subgroups of orthologous genes. PhyloTreePruner will not identify any subgroups of single-copy orthologous genes. (B) OrthoSNAP will identify 5 subgroups of single-copy orthologous genes (light blue) by identifying maximally inclusive subgroups—subtrees where each taxon is represented by a single sequence—and maximally inclusive subgroups after species-specific inparalog trimming (species-specific inparalogs are shown in orange). In contrast, DISCO and maximally inclusive strategies will identify 3 SC-OGs, in part, because they do not account for species-specific inparalogs. PhyloTreePruner, which only prunes species-specific inparalogs, will not identify any subgroups of single-copy orthologous genes due to the presence of more ancient duplication events. (TIF) [file pbio.3001827.s008.tif]
