# Supplementary material for: OrthoSNAP: A tree splitting and pruning algorithm for retrieving single-copy orthologs from gene family trees
Source: PLoS Biol. 2022 Oct 13;20(10):e3001827. doi: 10.1371/journal.pbio.3001827 (PMC9595520; doi:10.1371/journal.pbio.3001827)
Supplement: Attachment — Submitted filename: response_to_reviewers_orthosnap.docx [file pbio.3001827.s019.docx]

**Editor:**

1) Thank you for submitting your manuscript "orthoSNAP: a tree splitting and pruning algorithm for retrieving single-copy orthologs from gene family trees" for consideration as a Methods and Resources at PLOS Biology. Your manuscript has been evaluated by the PLOS Biology editors, an Academic Editor with relevant expertise, and by three independent reviewers.

You'll see that each of the reviewers is broadly positive about your study, but each raises a number of concerns that need to be addressed before further consideration. For example, you’ll see that Reviewer #1 wants you to clarify how the method differs from others, Reviewer #2 worries that your method is simplistic and might break down when faced with more complex scenarios (and wants to be convinced otherwise), and Reviewer #3 found orthoSNAP easy to use, but queries the decision to always retain the longest orthologue, questions the power of your stats, and wonders whether you could incorporate synteny.

In light of the reviews (below), we will not be able to accept the current version of the manuscript, but we would welcome re-submission of a much-revised version that takes into account the reviewers' comments. We cannot make any decision about publication until we have seen the revised manuscript and your response to the reviewers' comments. Your revised manuscript is also likely to be sent for further evaluation by the reviewers.

**We thank the editor for handling our manuscript and for guidance on the constructive comments raised by the reviewers. By modifying our algorithm and running several different analyses on additional phylogenomic datasets (hence the substantial delay in submitting this revision), we believe we have addressed all reviewers’ comments, greatly improving the overall quality and clarity of our manuscript.**

**Importantly, we have addressed all major points raised by the reviewers.**

**For Reviewer #1, we have provided additional text, tables, and figures describing how OrthoSNAP differs from algorithms, such as PhyloTreePruner, Agalma, DISCO, and OMA. These additions have clarified how – by conducting species-specific inparalog trimming and, as noted by reviewers, ease of use – OrthoSNAP is an improvement over existing algorithms.**

**For Reviewer #2, we built and examined three new datasets representing complex scenarios and examined the ability of OrthoSNAP to identify SNAP-OGs. Specifically, the reviewer requested that we test the OrthoSNAP workflow on a dataset of budding yeasts wherein half have experienced a whole genome duplication event as well as plants, which have complex histories of gene duplication and loss. We have included analyses of these datasets. Furthermore, we included an additional dataset of transcriptomes, which, unlike the previous two, have artificially high numbers of paralogs (e.g., several alternatively spliced transcripts are incorrectly inferred as paralogs). Our initial submission featured analyses of 46,645 orthologous groups of genes, however, the inclusion of these three new datasets resulted in the analysis of 415,129 total orthologous groups of genes representing an 8.9-fold increase. Across all datasets, we identified 9,821 SC-OGs and 10,704 SNAP-OGs. Analyses among the additional SC-OGs and SNAP-OGs corroborated and strengthened our previous findings suggesting, for example, OrthoSNAP is useful for identifying additional markers for phylogenetic and molecular evolution analyses.**

**For Reviewer #3, whose primary concern was keeping the practice of keeping the longest sequence in the case of species-specific inparalogs, we have now added substantial user flexibility for which sequence to keep. Specifically, users can now add an additional argument to specify whether the longest, shortest, or median (in the case of three or more sequences) length sequence should be retained. Furthermore, users can also choose which sequence to keep based on branch length (longest, shortest, or median in the case of three or more sequences). This allows fine control of species-specific inparalog choice for users. Lastly, we discuss incorporating synteny (and other information such as sequence similarity) into future iterations of OrthoSNAP.**

**Taken together, the revised manuscript represents a substantial effort that includes both algorithm modification and additional analyses. This effort has strengthened our previous findings and demonstrated OrthoSNAP can successfully identify SNAP-OGs in complex scenarios. We hope that the revised manuscript now merits publication in *PLOS Biology*.**

**Reviewer #1:**

**[identifies himself as Yannis Nevers]**

1) The authors present OrthoSNAP, a method that automatically extracts marker genes (a set of orthologous sequences with representatives from most species in a given dataset) from a set of gene families and their corresponding gene trees.

Contrary to more traditional method; it does not focus on single copy orthologous groups (sc-OGs) and also extracts orthologous groups from gene families even in presence of duplication (termed SNAP-OGs). Using SNAP-OGs, and not only Sc-OG, allows to increase the number of gene markers one can extract from a set species, to later use in phylogenomic analyses.

The authors show, comparing it over several descriptors, that the phylogenetic signal in SNAP-OGs is not significantly different than one in Sc-OG. They also show in a specific case that using SNAP-OGs for species tree determination on a difficult to infer taxonomic node yield similar results than using sc-OG, suggesting SNAP-OGs could be used the same way sc-OGs are. The analyses are convincing and to my knowledge, one of the first confirmation that SNAP-OGs can be used on the same capacity of Sc-OGs in phylogenomic analysis.

The manuscript is well organized and clearly written. My review below is divided into sections relative to different aspects of the work, and concludes with my recommandations.

**We thank the reviewer for their careful reading of our manuscript. We too are excited by our finding that SNAP-OGs harbor similar phylogenetic information compared to SC-OGs suggesting they are useful for broad downstream analyses, including phylogenomics.**

NOVELTY:

1) SNAP-OG is based on a tree splitting and pruning strategy, used to isolate OGs nested into gene families that underwent ancient duplications and keep only one copies from recently duplicated species-specific inparalog. Generally, the aim is to extract a set of orthologous sequences with only one representative of most of the species of interest, even in presence of duplication events.

As the authors discuss in the introduction, this is not per se new in concept. In particular, methods like PhyloTreepruner and Agalma have similar aims and can at least be used to handle species-specific inparalogs.

The authors discuss this and claim their method handles more types of paralogy than previously existing methods, and to my understanding, can extract more OGs from a set of gene families with duplication events than these methods could (presumably because of the combination of tree splitting and pruning that is implemented in OrthoSNAP). Still, it is unclear from the current discussion exactly how they differ, and in what concrete case OrthoSNAP yields more orthologous groups than these methods.

**We thank the reviewer for their comment and apologize for the lack of clarity. We have expanded text in the discussion to detail that OrthoSNAP differs from PhyloTreePruner and Agalma because it can conduct both in-paralog trimming and identifying subtrees of single-copy orthologous genes. In contrast, PhyloTreePruner can only conduct in-paralog trimming and Agalma can only identify maximally inclusive subtrees. As a result, OrthoSNAP, at least in some instances, will be able to identify SNAP-OGs across more gene families than either PhyloTreePruner or Agalma.**

**Another important difference is the input for each algorithm. Specifically, Agalma and PhyloTreePruner both require rooted phylogenies; in contrast, OrthoSNAP will automatically midpoint root inputted phylogenies; however, users can also input pre-rooted phylogenies using a new parameter. This allows for greater user flexibility in the OrthoSNAP framework.**

**In addition to the expanded text, we have also included a figure that depicts how each algorithm would handle the test case highlighted in Figure 1B. In this case, OrthoSNAP identifies more subgroups of single-copy orthologous genes compared to the maximum inclusion strategy (as implemented in Agalma), DISCO, and PhyloTreePruner methods. We have also created a supplementary table that summarizes the similarities and differences between methods that identify subgroups of single-copy orthologous groups of genes using tree-based methods (OrthoSNAP, maximum inclusion (as implemented in Agalma), DISCO, and PhyloTreePruner) and graph-based methods (OMA).**

2) Additionally, two other published works appear to implement similar strategies to extract OGs from gene families with duplication: DISCO (Willson et al, 2021) [doi: 10.1093/sysbio/syab070] and the different strategies of orthologs sampling proposed in Yang and Smith, 20145] [doi:10.1093/molbev/msu24 (in particular the Maximum inclusion strategy). These works are cited in the manuscript but there is no discussion relative to the strategy they use for what looks like to be a similar task.

**We thank the reviewer for their comment. DISCO (Wilson et al. 2021, Systematic Biology) conducts multi-copy gene tree decomposition, which prioritizes creating at least one large single-copy gene tree while also retaining single-copy gene subtrees resulting in some subtrees that are combinations of single representatives of paralogs and “truly” single-copy genes. In contrast, OrthoSNAP does allow combinations of single representatives of paralogs and “truly” single-copy genes to be part of the same SNAP-OG.**

**We thank the reviewer for pointing out that we have inadequately described the “maximum inclusion strategy” from Yang and Smith, 2014. The maximum inclusion strategy is the same as the one implemented in Agalma, which aims to identify the subtree with the highest number of taxa represented by a single sequence. We note that the maximum inclusion strategy fails to identify subgroups of single-copy orthologous genes in transcriptome datasets where the number of transcripts greatly exceeds the number of protein coding genes present in the genome. In such cases, the strategy tends to infer groups that contain a mix of orthologs and paralogs.**

**Furthermore, as the reviewer noted, we evaluated the phylogenetic information content of SNAP-OGs, which has not been done previously. However, the DISCO team did demonstrate the utility of their tree decomposition algorithm for use in coalescence- or quartet-based species tree estimation (i.e., using ASTRAL). We have expanded on text that further elucidates these similarities and differences as well as acknowledges utility of SNAP-OGs identified by OrthoSNAP for coalescence- or quartet-based species tree estimation or examination of phylogenetic signal. To facilitate downstream coalescence-based species tree estimation, OrthoSNAP now outputs Newick tree files of SNAP-OGs.**

**Lastly, we have also included an additional supplementary figure that compares the different algorithms using the same phylogenetic tree featured in figure 1B.**

3) Finally, I note that SNAP-OGs provided by OrthoSNAP seems to be conceptually similar to OMA Groups provided by the OMA Orthology inference method (see Identifying orthologs with OMA: A primer, Wahn-Zabal et al, doi: 10.12688/f1000research.21508.1). Do the authors believe it is the case? If so, it may be worth mentioning.

**We thank the reviewer for bringing this article to our attention. We agree that it is worth mentioning. In the revised manuscript, we note that both OrthoSNAP and OMA are conceptually similar in that they both aim to identify subgroups of orthologs within larger groups of orthologous genes. A key difference is that OMA uses sequence similarity and graph-based clustering to identify orthologs and subgroups therein whereas OrthoSNAP uses phylogenetic trees of orthologous groups of genes. Thus, when discussing practical considerations when using OrthoSNAP, we also note that OMA, like OrthoMCL, can be used upstream of OrthoSNAP.**

**As noted in our response to comment #1, we have included a table that summarizes the similarities and differences among these pieces of software.**

4) While I believe these aspects may be discussed a bit more (see recommendations), OrthoSNAP does seem to implement a different strategy than existing methods and the fact that it is implemented as an user-friendly command line tool with few input constraints is valuable.

**We thank the reviewer for seeing the merit and value of our study. We are excited by the toolkit we have developed, and hope others will find it of use for their molecular evolution studies.**

5) The assessment of the difference between sc-OGs and SNAP-OGs is both new and has interesting implications regarding the use of SNAP-OGs in phylogenomic studies. It has a similar conclusion than another independent but somewhat similar preprinted studies. This is also addressed by the authors in the discussion section.

**We thank the reviewer for their comment.**

SOFTWARE ACCESSIBILITY, EASE OF USE AND REPRODUCIBILITY

6) The code is available on GitHub, and is well documented. The documentation provided an easy and quick way to install the software and was sufficient for me to successfully run it on a test dataset.

The datasets used in the course of the work are freely available on figshare and the protocole is sufficiently described in the method section

**We thank the reviewer for their comment. We believe it is of utmost importance to conduct science transparently and reproducibly. We believe OrthoSNAP exemplifies our stance on transparent and reproducible science. This in part includes our freely available version-controlled code on GitHub as well as extensive documentation that comes complete with tutorials.**

RECOMMENDATION:

7) I believe the work would be of interest for the community, in good part because it provides easy to use software for the extraction of marker genes from phylogenomic datasets, even in presence of duplication. I thus recommend its publication without additional analyses needed.

However, given the similarity of the work with previously available software, I'd like to see a more detailed discussion on how OrthoSNAP differs from previous works. In particular:

-The author indicates that OrthoSNAP is different from two other tree pruning protocole. It would be useful to indicate a few example cases of when it is expected to provide different results.

-I would also be interested in a discussion of the comparison with other published strategies for what seems to be a similar task: in particular the Maximum inclusion strategy from Yang et al, 2014, the methods used by DISCO. (See the Novelty section above)

**We thank the reviewer for their comment and for seeing the value of our work. Given that this comment is similar to comments #2 and #3, we have now expanded on the comparison between orthoSNAP and other similar algorithms greatly in the revised manuscript. We have also created supplementary data (one figure; one table) that summarize the similarity and differences between OrthoSNAP, DISCO, PhyloTreePruner, Maximum inclusion, and OMA.**

Minor remark:

-The authors use the word "taxon" to refer only to the individual species in their datasets. It causes a bit of confusion because usually any grouping of multiple species sharing a common ancestor may be considered a taxon. I suggest the authors disambiguate the term.

**We thank the reviewer for their comment. We have revised our use of taxon in the manuscript.**

**Reviewer #2:**

1) The authors present here orthoSNAP a program designed to obtain orthogroups from gene trees including duplications with the objective of obtaining more marker genes and therefore increasing the number of genes available for numerous evolutionary studies. As stated by the authors, this has been done before in multiple ways and the improvement given by orthoSNAP is more focused on usability by non-expert users than on creating an innovative algorithm. They then test their orthoSNAP on different sets of species: budding yeasts, filamentous fungi and a set of mammals. They show how using orthoSNAP they can increase the number of orthogroups significantly depending on the dataset and that those orthogroups are not different in terms of general phylogenetic characteristics making them suitable for posterior analyses.

**We thank the reviewer for their comments on our manuscript. We have addressed all comments as described below. We believe that our manuscript has substantially improved in terms of clarity and rigor. Additionally, we have expanded the number of user options, increasing our program’s utility and user-friendliness.**

2) I have concerns that the orthoSNAP algorithm is a bit too simple and will not hold up in the case of more complex scenarios (note also my comments on the datasets below). A very obvious scenario in which I think orthoSNAP will fail is when duplications are concentrated in a given node. For instance, imagine that the budding yeast dataset was formed of more than 50% species that underwent the WGD that happened in the Saccharomyces cerevisiae lineage and the rest was made of pre-WGD species. As I understand, the algorithm would look for clades that include more than 50% of the species set and no duplications after removing species specific duplicates. In the dataset I proposed you would have numerous orthogroups that would include uniquely post-WGD species and would not include the pre-WGD orthologs despite existing, being present in the tree and being easily detectable. I wonder if the taxon distribution between SC-OGs and SNAP-OGs would still be the same and how the other metrics would be affected. The same problem can be extended to any tree analysed, orthologs found before a duplication large enough to cope the taxon occupancy threshold will not be included in orthogroups.

**We thank the reviewer for their comment. We agree that using a strict threshold for the percentage of species that must be present in a SNAP-OG can be problematic in certain scenarios. That is why OrthoSNAP has the -o/--occupancy argument that allows users to specify an occupancy threshold for a SNAP-OG. By default, the occupancy threshold is 50%, however, users may input a specific integer value to use as an occupancy threshold. Thus, if a user inputs “4” with the -o/--occupancy argument, OrthoSNAP will set the minimum threshold of a SNAP-OGs to be four species. With this and other parameters in OrthoSNAP, researchers can fine tune parameters to what best fits their research scenario or question.**

**We also explored three more complex evolutionary scenarios: 1) a dataset comprised of half pre- and half post-whole genome duplication (WGD) budding yeast species, 2) a dataset of plants, which have a complex history of duplication and loss, and 3) a transcriptome dataset, which has a much higher number of transcripts than genes (because often many transcripts stem from a single gene). Details of these results, as well as the datasets, are provided in our response to the next comment—comment #3.**

3) Note that in the introduction the authors used plants as an example of a complicated dataset where tools such as orthoSNAP would be of great advantage, yet the authors used two fungal datasets for which they already have a significant amount of single copy families. This is great for showing whether there are differences between SC-OGs and SNAP-OGs, but they do not offer a true challenge for the program. The mammal dataset is more meaningful in terms of what orthoSNAP can offer and I think there should be more emphasis put on this.

**We thank the reviewer for their comment. We agree that plants would be a great dataset to demonstrate the utility of OrthoSNAP. Thus, we ran the OrthoSNAP workflow one a dataset of 30 plants. To further challenge the program, we ran the OrthoSNAP workflow on a dataset of 20 transcriptomes from choanoflagellates, whose genes are known to often correspond to multiple transcripts, and 30 budding yeast species wherein 15 have undergone whole genome duplication and 15 have not. An additional supplementary table has been generated to summarize these datasets. We also provide a main text table that summarizes these findings. The table is also shown here for clarity.**

| ***Dataset*** | ***Reason for difficulty*** | ***Total OGs*** | ***SC-OGs***  ***(50% min. occupancy threshold)*** | ***SNAP-OGs (50% min. occupancy threshold)*** | ***SC-OGs***  ***(4-taxon min. threshold)*** | ***SNAP-OGs (4-taxon min. threshold)*** |
| --- | --- | --- | --- | --- | --- | --- |
| *Plants (N=30)* | *Evolutionary histories with extensive gene duplication and loss events* | *83,034* | *15* | *653* | *200* | *15,854* |
| *Budding yeasts (N=30)* | *Half of the species used experienced whole-genome duplication* | *11,422* | *2,782* | *1,334* | *3,566* | *4,199* |
| *Choanoflagellates (N=20)* | *Transcriptomes, where often multiple transcripts correspond to a single protein-coding gene* | *274,028* | *390* | *2,087* | *2,438* | *11,556* |

***OrthoSNAP successfully identifies SNAP-OGs in challenging datasets such as those with complex evolutionary histories (plants with many gene duplication and loss events; budding yeasts that have undergone a whole-genome duplication event) and transcriptomic data.***

**These findings demonstrate that OrthoSNAP holds promise for identifying SNAP-OGs in “difficult” datasets such as those that are composed of species with complex evolutionary histories (like plants and the whole-genome duplication in budding yeast) as well as having an artificially high number of duplicated genes (like in transcriptomic data). Thus, OrthoSNAP is helpful for identifying subgroups of orthologous genes for molecular evolutionary studies both in the presence of real duplicates (e.g., those that stem from ancestral duplication events) and in the presence of technical/artificial duplicates (e.g., multiple transcripts stemming from a single gene).**

4) Regarding the mammal dataset, the improvement is notable and it certainly is useful but I miss a bit more of context on why of the 17407 multi copy families orthoSNAP only retrieves 1775.

**We thank the reviewer for their comment. Although OrthoSNAP was able to identify 1,775 SNAP-OGs, we note that this is a substantial improvement from the original number of SC-OGs, 321. Thus, the total number of orthologs available for molecular evolution analyses has increased over five-fold. There are three reasons that contribute to why SNAP-OGs could not be identified among some genes and we have now included text to describe them in the revised manuscript. These are: 1) gene families with sequence data from <50% of the taxa, 2) gene families with complex evolutionary histories (e.g., HGT, duplication/loss patterns), and 3) gene families with evolutionary histories that differ from the species tree (e.g., due to analytical factors, such as sampling and systematic error, or biological factors, such as lineage sorting or introgression). Notably, the first reason can, but does not always, result in inability to infer SNAP-OGs and can be, to a certain extent, addressed (e.g., by lowering the orthogroup occupancy threshold in OrthoSNAP), whereas the other two reasons are more challenging because they often result in a genuine absence of single-copy orthologs. Furthermore, the actual number of single-copy orthologs is not known, making it challenging to infer whether there are many other putative orthologs that are being missed or not. This issue has long challenged researchers, even when employing genome synteny to infer orthology (**[**https://www.nature.com/articles/nature04562**](https://www.nature.com/articles/nature04562)**). Lastly, we note that users may change the occupancy threshold based on their needs and that lower SNAP-OG occupancy thresholds will lead to more SNAP-OGs being identified.**

5) Regarding the comparison between SC-OGs and SNAP-OGs, I understand that the underlying point of the analysis is to show that orthogroups build from single copy genes and from SNAP will give similar results and that it is so because orthoSNAP is able to successfully capture only orthologs and not include unwanted paralogs. For that point to be meaningful, you should also show that randomly including paralogs would be detrimental to the metrics you are using and would therefore give a different signal in these specific datasets. If not added, one could argue that, no matter how well or poorly SNAP performs the results are always going to be the same. Points also to see whether mixing different kinds of paralogs have different effects.

**We thank the reviewer for their comment. We note that the main objective of comparing SC-OGs and SNAP-OGs was to determine whether they harbor similar phylogenetic information. To date, other similar algorithms have not examined the phylogenetic features of the subtrees identified. Typically, other software or analyses (such as the one described in the DISCO software) demonstrate that they are be used for phylogenomics without demonstrating if they are *fit* to be used for phylogenomics. Our finding that the phylogenetic information content of SC-OGs and SNAP-OGs is similar suggests SNAP-OGs identified using OrthoSNAP are fit for use in phylogenomics.**

**Notwithstanding these observations, we agree that constructing groups of genes that are combinations of orthologs and paralogs will help elucidate the phylogenetic informativeness of SNAP-OGs. To examine the similarities and differences between SC-OGs, SNAP-OGs, and random combinations of orthologs and paralogs, we first constructed random combinations of orthologs and paralogs (hereafter referred to as Random-GGs for *random* combinations of orthologous and paralogous *g*roups of *g*enes) in the budding yeast (N=647), filamentous fungi (N=999), and mammalian (N=954) datasets. To do so, a single sequence was randomly selected from representative species in multi-copy orthologous genes. We then aligned, trimmed, and inferred phylogenetic trees among the Random-GGs. Following the results in figure 3, information content among the alignments and phylogenetic trees inferred among Random-GGs.**

**Random-GGs are significantly different from SC-OGs and SNAP-OGs. Specifically, across each dataset, significant differences were observed among SC-OGs, SNAP-OGs, and Random-GGs (p < 0.001, F = 189.92, df = 4; Multi-factor ANOVA). Further examination of differences revealed Random-GGs are significantly different compared to SC-OGs and SNAP-OGs (p < 0.001 for both comparisons; Tukey honest significant differences (THSD) test) in the budding yeast dataset. In contrast, SC-OGs and SNAP-OGs are not significantly different (p = 0.42; THSD). The same was also true for the dataset of filamentous fungi and mammals—specifically, Random-GGs are significantly different from SC-OGs and SNAP-OGs (p < 0.001 for each comparison in each dataset; THSD), whereas SC-OGs and SNAP-OGs are not significantly different (p = 1.00 for filamentous fungi dataset; p = 0.42 for mammals dataset; THSD).**

**Further examination of the drivers differentiating Random-GGs from SC-OGs and SNAP-OGs using principal component (PC) analysis revealed Robinson-Foulds distances (RF distance; a measure of tree accuracy wherein lower values represent greater tree accuracy), and relative compositions variability (RCV; a measure of alignment composition bias wherein lower values represent lower compositional bias), often drove differences among Random-GGs, SC-OGs, and SNAP-OGs across the datasets. Specifically, in PC space, the z-score contribution of RF distance along the first dimension for the budding yeast dataset is 0.74 and for the filamentous fungi dataset is 0.89. The z-score contribution of RF distance along the second dimension for the mammalian dataset is 1.57. Therefore, we further compared the mean of RF distances and RCV values.**

| ***Dataset*** | ***OG type*** | ***RF distance*** | ***RCV*** |
| --- | --- | --- | --- |
| *Budding yeasts* | *SC-OGs* | *0.19 ± 0.12* | *0.19 ± 0.05* |
|  | *SNAP-OGs* | *0.18 ± 0.11* | *0.18 ± 0.06* |
|  | *Random-GGs* | ***0.65 ± 0.27*** | ***0.27 ± 0.13*** |
| *Filamentous Fungi* | *SC-OGs* | *0.27 ± 0.13* | *0.12 ± 0.05* |
|  | *SNAP-OGs* | *0.27 ± 0.12* | *0.12 ± 0.06* |
|  | *Random-GGs* | ***0.87 ± 0.11*** | ***0.21 ± 0.13*** |
| *Mammals* | *SC-OGs* | *0.56 ± 0.22* | *0.13 ± 0.06* |
|  | *SNAP-OGs* | *0.51 ± 0.23* | *0.11 ± 0.07* |
|  | *Random-GGs* | ***0.61 ± 0.30*** | ***0.15 ± 0.10*** |

**In each case, the Random-GGs had worse RF distance and RCV values (indicated in bold). These differences were more pronounced in the datasets of budding yeasts and filamentous fungi. Taken together, these results suggest that SC-OGs and SNAP-OGs have similar information content, but this content significantly differs from that in random combinations of orthologs and paralogs.**

Minor comments:

6) When having a species specific duplication orthoSNAP keeps only the gene with the longest sequence, argument based on transcriptomic data. I would be interested in reading why authors did not choose to keep the paralog with the shortest branch as that would be more conservative when thinking on running some of the posterior analyses.

**We thank the reviewer for their comment. We have chosen to keep the longest sequence because, in the case of transcriptomic data, is likely the most complete splice variant. We also note that Kocot et al. (doi: 10.4137/EBO.S12813) also advocated for this approach. However, we agree that the longest sequence may not always be the most appropriate. We also acknowledge that is likely impossible to develop one “best” approach for all cases of in-paralogs and for all downstream analyses. Thus, we have included an additional argument that enables users to pick if the longest, median (in the case of three or more species-specific inparalogs), or shortest sequence should be picked. Alternatively, as recommended by the reviewer, branch lengths may be a helpful way to select which species-specific inparalog to keep. Thus, users can also specify if the species-specific inparalog with the longest, median (in the case of three or more species-specific inparalogs), or shortest distance (measured in tip-to-root substitutions per site) should be selected. Thus, we have provided substantially more user flexibility in OrthoSNAP.**

7) In the results section I miss having plain numbers shown about the real improvement of orthoSNAP. It is put as a percentage and directed to a supplementary table but I think the number of orthogroups retrieved for each dataset should be put there.

**We thank the reviewer for their comment and have included the plain numbers in the results section when reporting how many SC-OGs and SNAP-OGs there are.**

8) It would also be interesting to have more of an idea from the start which species are used in the analysis. Filamentous fungi is not the same as saying a group of Aspergillus and Penicillium genomes that are very closely related.

**We thank the reviewer for their comment and have now expanded the in-text description of the datasets. For example, we explicitly state that the dataset of filamentous fungi exclusively features *Aspergillus* and *Penicillium* species.**

9) I wonder how orthoSNAP would perform without having the pre-filter of orthofinder. Orthofinder is already putting genes into orthologous families but one could build a tree based on blast results and just run orthoSNAP on that. Would results be comparable?

**We thank the reviewer for their comment. We have expanded the discussion section to demonstrate how OrthoSNAP can be used in tandem with other software—such as BLAST. Broadly, we expect results to be comparable, however, they will be largely influenced by the sequences used for tree inference and the resolution therein. We have expanded our text in the discussion that underscores the impact of upstream analyses on inferences made by OrthoSNAP.**

**Reviewer #3:**

**[identify themselves as Giulio Formenti and Erich Jarvis]**

General comments:

1) Steenwyk et al. present orthoSNAP, a tree splitting and pruning algorithm for retrieving single-copy orthologs from gene family trees. The tool is aimed at addressing the important issue in comparative genomics of identifying unique orthologous genes for evolutionary inference.

**We thank the reviewers for their careful reading of the manuscript and constructive feedback. We have addressed all their comments below. In doing so, the overall quality of the manuscript and OrthoSNAP has improved.**

2) The logic behind the tool is clear, well-explained, and well-detailed. The tool is well-documented, and we were easily able to install it (on Mac) and run it using the test data set and the tutorial provided. The authors also use orthoSNAP to explore two contentious alternative hypotheses of deep evolutionary relationships among placental mammals. The good news is that from 46,645 orthologous groups of genes, their method identified 6400 more single copy orthologs in addition to the 6600 by another method. My worrisome news is what the many other putative ortholos of the ~46,000?. Perhaps we am missing something here?

**We thank the reviewers for their comment. We have expanded in the discussion that families of orthologous genes wherein no SNAP-OG were identified may have complex patterns of duplication and loss or be too poorly resolved to robustly identify a SNAP-OG. Despite numerous orthologous groups of genes not being suitable for identifying SNAP-OGs in the mammalian dataset, we note that OrthoSNAP substantially increased the mammalian dataset by 5.53-fold. It should also be noted that the actual number of single-copy orthologs is not known, making it challenging to infer whether there are many other putative orthologs that are being missed or not. What we can evaluate is that OrthoSNAP is able to—at least in some instances—greatly increase the size of datasets.**

**However, we do note at least one way that users can obtain more SNAP-OGs. Specifically, modifying the value used for the -o/--occupancy argument. By default, the occupancy threshold is 50%, however, users may input a specific integer value to use as an occupancy threshold. Thus, if a user inputs “4” with the -o/--occupancy argument, OrthoSNAP will set the minimum threshold of a SNAP-OGs to be four species, a quartet. With this and other arguments in OrthoSNAP, researchers can fine tune parameters to what best fits their research scenario or question.**

**To demonstrate how modifying this parameter can greatly change the number of SNAP-OGs identified, we tested OrthoSNAP on multiple datasets. Specifically, these datasets (N=3) also test the efficacy of OrthoSNAP under complex evolutionary and artifactual scenarios—a recommendation made by reviewer #2. Specifically, one dataset was composed of half pre- and half post-whole genome duplication budding yeast species. Another dataset was composed of plants, which have a complex history of duplication and loss. Lastly, a third data was composed of transcriptomes, which typically have multiple transcripts per protein coding genes.**

**Corroborating previous findings, OrthoSNAP can greatly expand the number of single-copy orthologs for molecular evolutionary analyses. Furthermore, by changing the -o/--occupancy parameter to “4”, we were able to identify substantially more SNAP-OGs. The following table summarizes these results.**

| ***Dataset*** | ***Reason for difficulty*** | ***Total OGs*** | ***SC-OGs***  ***(50% min. occupancy threshold)*** | ***SNAP-OGs (50% min. occupancy threshold)*** | ***SC-OGs***  ***(4-taxon min. threshold)*** | ***SNAP-OGs (4-taxon min. threshold)*** |
| --- | --- | --- | --- | --- | --- | --- |
| *Plants (N=30)* | *Evolutionary histories with extensive gene duplication and loss events* | *83,034* | *15* | *653* | *200* | *15,854* |
| *Budding yeasts (N=30)* | *Half of the species used have undergone whole-genome duplication* | *11,422* | *2,782* | *1,334* | *3,566* | *4,199* |
| *Choanoflagellates (N=20)* | *Transcriptomes, which have more transcripts than protein coding genes* | *274,028* | *390* | *2,087* | *2,438* | *11,556* |

***OrthoSNAP successfully identifies SNAP-OGs in challenging datasets such as those with complex evolutionary histories (plants with many gene duplication and loss events; budding yeasts that have undergone a whole-genome duplication event) and transcriptomic data.***

**These findings demonstrate that OrthoSNAP holds promise for identifying SNAP-OGs in “difficult” datasets such as those that are composed of species with complex evolutionary histories (like plants and the whole-genome duplication in budding yeast) as well as having an artificially high number of duplicated genes (like in transcriptomic data). Thus, OrthoSNAP is helpful for identifying subgroups of orthologous genes for molecular evolutionary studies both in the presence of real duplicates (e.g., those that stem from ancestral duplication events) and in the presence of technical/artificial duplicates (e.g., multiple transcripts stemming from a single gene).**

**In summary, there are three reasons that contribute to why SNAP-OGs could not be identified among some genes and we have now included text to describe them in the revised manuscript. These are: 1) gene families with sequence data from <50% of the taxa, 2) gene families with complex evolutionary histories (e.g., HGT, duplication/loss patterns), and 3) gene families with evolutionary histories that differ from the species tree (e.g., due to analytical factors, such as sampling and systematic error, or biological factors, such as lineage sorting or introgression). Notably, the first reason can, but does not always, result in inability to infer SNAP-OGs and can be, to a certain extent, addressed (e.g., by lowering the orthogroup occupancy threshold in OrthoSNAP), whereas the other two reasons are more challenging because they often result in a genuine absence of single-copy orthologs. Furthermore, the actual number of single-copy orthologs is not known, making it challenging to infer whether there are many other putative orthologs that are being missed or not. This issue has long challenged researchers, even when genome synteny is used to infer orthology (**[**https://www.nature.com/articles/nature04562**](https://www.nature.com/articles/nature04562)**). Lastly, we note that users may change the occupancy threshold based on their needs and that lower SNAP-OG occupancy thresholds will lead to more SNAP-OGs being identified.**

3) Besides this, we have three main concerns, one regarding the simplicity of a key step of the pipeline, another regarding statistics, and a third regarding complementary evidence, such as synteny.

**We thank the reviewers for their comments and have addressed them below.**

4) With respect to simplicity:

At the pruning step, the paralog to be retained is always the longest. The justification given for this choice is that it is common practice in transcriptomics. I can see why this is the case in transcriptomics, e.g. to increase mappability to the genome, however in the alignment of ortholog genes, choosing the longest transcript this may not be the best choice and could rather introduce bias in the results to a particular spliced exon missing in some of the longest transcript or additional erroneously annotated codons. I wonder if this very simple assumption could undermine some of the results. Can complementary/alternative method using all transcripts, be envisaged and introduced?

**We thank the reviewers for their comment, which is similar to comment #6 by reviewer #2. We chose to keep the longest sequence because, in the case of transcriptomic data, is likely the most complete splice variant. Kocot et al. (doi: 10.4137/EBO.S12813) also advocated for this approach. However, we agree that the longest sequence may not always be the most appropriate. Thus, we have included an additional argument that enables users to pick if the longest, median (in the case of three or more species-specific inparalogs), or shortest sequence should be picked. Alternatively, as recommended by the reviewer, branch lengths may be a helpful way to select which species-specific inparalog to keep. Thus, users can also specify if the species-specific inparalog with the longest, median (in the case of three or more species-specific inparalogs), or shortest distance (measured in tip-to-root substitutions per site) should be selected. Thus, we have substantially increased the user flexibility of OrthoSNAP.**

5) With respect to statistics:

Throughout the manuscript, the absence of statistical significance is generally given as evidence to conclude that identified SNAP-OGs behave similarly to SG-OGs. Since the absence of significance can be due to no real significance or not enough power, these results and their interpretation should be phrased more carefully. Additionally, to some degree, one would expect genes with high numbers of paralogs to behave somewhat differently from truly single-copy orthologs; there seems to be some evidence that this is happening, which would be interesting to characterize since, as the authors put it, "the phylogenetic information content of these gene families remains unknown". In particular:

-	It seems that the non-significant results in the multivariate analyses may be partially explained by the high dispersion of the data, including in at least some cases the presence of many outliers (e.g. in alignment length, parsimony informative sites, RCV and treeness show higher SD in mammalian SNAP-OGs Fig S9). Are there at least some genes that behave differently? Can the authors comment on that? For instance, in Budding yeast it seems that tree certainty is consistently lower in SNAP-OGs vs SG-OGs (ST6). Can the authors further describe ST6 and comment on this in the main text?

**We thank the reviewers for their comment. We have included text in the results section that reports differences in the shapes of some distributions (as noted by the reviewers) despite the lack of statistical significance. We also acknowledge that despite the lack of significant differences between SNAP-OGs and SC-OGs, further comparative analysis, especially in different lineages, will further our understanding of SNAP-OGs.**

**Notwithstanding these observations, following a suggestion from reviewer #2, we compared SC-OGs and SNAP-OGs groups of genes that are combinations of randomly selected orthologs and paralogs (hereafter referred to as Random-GGs for *random* combinations of orthologous and paralogous *g*roups of *g*enes). Thereafter, we examined the similarities and differences between SC-OGs, SNAP-OGs, and Random-GGs. In brief, our findings corroborated previous reports that SC-OGs and SNAP-OGs are not different from one another, whereas Random-GGs are. These findings support the idea that SNAP-OGs are useful for phylogenomics and molecular evolution analysis. Further details (including quantitative analysis) are provided below.**

**To examine the similarities and differences between SC-OGs, SNAP-OGs, and Random-GGs, we first constructed random combinations of orthologs and paralogs in the budding yeast (N=647), filamentous fungi (N=999), and mammalian (N=954) datasets. To do so, a single sequence was randomly selected from representative species in multi-copy orthologous genes. We then aligned, trimmed, and inferred phylogenetic trees among the Random-GGs. Following the results in figure 3, information content among the alignments and phylogenetic trees inferred among Random-GGs.**

**Random-GGs are significantly different from SC-OGs and SNAP-OGs. Specifically, across each dataset, significant differences were observed among SC-OGs, SNAP-OGs, and Random-GGs (p < 0.001, F = 189.92, df = 4; Multi-factor ANOVA). Further examination of differences revealed Random-GGs are significantly different compared to SC-OGs and SNAP-OGs (p < 0.001 for both comparisons; Tukey honest significant differences (THSD) test) in the budding yeast dataset. In contrast, SC-OGs and SNAP-OGs are not significantly different (p = 0.42; THSD). The same was also true for the dataset of filamentous fungi and mammals—specifically, Random-GGs are significantly different from SC-OGs and SNAP-OGs (p < 0.001 for each comparison in each dataset; THSD), whereas SC-OGs and SNAP-OGs are not significantly different (p = 1.00 for filamentous fungi dataset; p = 0.42 for mammals dataset; THSD).**

**Further examination of the drivers differentiating Random-GGs from SC-OGs and SNAP-OGs using principal component analysis revealed Robinson-Foulds distances (RF distance; a measure of tree accuracy wherein lower values represent greater tree accuracy), and relative compositions variability (RCV; a measure of alignment composition bias wherein lower values represent lower compositional bias), often drove differences among Random-GGs, SC-OGs, and SNAP-OGs across the datasets. Specifically, in PC space, the z-score contribution of RF distance along the first dimension for the budding yeast dataset is 0.74 and for the filamentous fungi dataset is 0.89. The z-score contribution of RF distance along the second dimension for the mammalian dataset is 1.57. Therefore, we further compared the mean of RF distances and RCV values**

| ***Dataset*** | ***OG type*** | ***RF distance*** | ***RCV*** |
| --- | --- | --- | --- |
| *Budding yeasts* | *SC-OGs* | *0.19 ± 0.12* | *0.19 ± 0.05* |
|  | *SNAP-OGs* | *0.18 ± 0.11* | *0.18 ± 0.06* |
|  | *Random-GGs* | ***0.65 ± 0.27*** | ***0.27 ± 0.13*** |
| *Filamentous Fungi* | *SC-OGs* | *0.27 ± 0.13* | *0.12 ± 0.05* |
|  | *SNAP-OGs* | *0.27 ± 0.12* | *0.12 ± 0.06* |
|  | *Random-GGs* | ***0.87 ± 0.11*** | ***0.21 ± 0.13*** |
| *Mammals* | *SC-OGs* | *0.56 ± 0.22* | *0.13 ± 0.06* |
|  | *SNAP-OGs* | *0.51 ± 0.23* | *0.11 ± 0.07* |
|  | *Random-GGs* | ***0.61 ± 0.30*** | ***0.15 ± 0.10*** |

**In each case, the Random-GGs had worse RF distance and RCV values (indicated in bold). These differences were more pronounced in the datasets of budding yeasts and filamentous fungi. Taken together, these results suggest that SC-OGs and SNAP-OGs have similar information content, which are significantly different from random combinations of orthologs and paralogs.**

6) -	In Fig 4B, can the authors explain why the added a third hypothesis when testing the evolutionary relationships in placental mammals ("Xenarthra as sister to all other Eutheria represented in yellow")? There is a discrepancy in the main text, as initially only two hypotheses are mentioned, but then a third is introduced and used for only one of the analyses. A third hypothesis will obviously reduce the statistical significance of the results after correction for multiple testing, potentially making results non-significant, which seems to be the case here. Also, why was the alpha set at 0.01 instead of the usual 0.05? Finally, do you think that more sophisticated statistical tests than Fisher's using GSF values that would be able to reveal differences?

**We thank the reviewers for their comment. We have now included in the main text all three alternative hypotheses and updated panel A in figure 4 to include all three topologies. Similarly, we redid the gene-wise log likelihood analysis considering the three possible topologies. This approach is more comprehensive because it captures all possible topologies of a rooted triplet. We have revised panel C to reflect analysis of gene support frequencies using likelihood analysis. Similar to topology-based tests (panel B), there is no difference between SC-OGs and SNAP-OGs (p = 0.52; Fisher’s exact test)**

**With respect to multiple test correction, we note that examining three hypotheses will not change the number of multiple test corrections that are conducted. The reasoning is that two tests were conducted—Fisher’s exact test comparing GSF values among SC-OGs and SNAP-OGs and SC-OGs and SNAP-OGs after collapsing poorly support bipartitions. We also note that an alpha of 0.01 and 0.05 will yield the same result. That is, a p-value of 0.05 is not less than 0.05; therefore, the GSF would still not be significant. Lastly, we note that our use of a Fisher’s exact test is not guided by “test sophistication”; instead, we chose the appropriate test for the given data and experimental set up. We have also confirmed that we used the appropriate test after consulting the textbook titled “The Analysis of Biological Data” by Michael C. Whitlock and Dolph Schluter.**

7) -	In Fig 4C, the outliers collapse most of the central points. However, the two distributions do look different. Aside from the absence of significant differences in GLS, which could be the result of the great dispersion of the data, could a test that compares the shape of the distribution reveal a significant difference? Also, Fig 4C should be bigger to better highlight the distribution? Maybe a transformation could also be applied to the y axis to better highlight the data.

**We thank the reviewers for their comment. Although panel C has substantially changed and now accounts for all three possible topologies, we explored transformations as per the reviewers’ request. Specifically, we explored a diverse panel of data transformations prior to plotting (see below). Specifically, we used a square, log, reciprocal, square, exponential, and median transformation. In the figure below, these are plotted from left to right and top to bottom after plotting with no transformation. The distributions were either visually comparable to no transformation (i.e., square root, log, and median transformations) or were more difficult to see (i.e., reciprocal, square, and exponential). We also redid the Wilcoxon Rank sum test for each data transformation. As expected, the results were the same. The sole exception was when the data was transformed by squaring each value (center tile in plot). However, the distributions are even more difficult to see. Therefore, we did not include this finding in the manuscript.**

8) With respect to synteny:

Because of different rates of difference, sometimes it is difficult to indentify orthologs versus paralogs based on sequence identity and phylogeny alone. Synteny is a third piece evidence that helps identify orthology. A good recent example on the oxytocin receptor and ligand family of genes (Theofanopoulou et al 2021 Nature). Have the authors utilized synteny? If not, it would be good to incorporate. At a minimum the authors should discuss this issue.

**We thank the reviewers for their comment. OrthoSNAP specifically uses phylogenetic trees to infer SNAP-OGs. We have expanded the discussion section of the manuscript to emphasize that orthologs predicted by all methods, including OrthoSNAP, are inferences; support for these inferences can be provided using several additional lines of evidence, including synteny. We also acknowledge that synteny will be difficult to infer for some lineages, such as budding yeasts, which are an ancient and rapidly evolving lineage with poorly conserved synteny (except in comparisons of very closely related species). Thus, OrthoSNAP can be helpful for instances of rapidly evolving taxa but may benefit from examination of synteny where possible.**

Specific comments:

Abstract:

9) - consider dropping "selection"", as negative selection also needs SC_OGs

**We thank the reviewers for their comment. We have removed the word “positive” to acknowledge that any surveys of selection—positive or negative—rely on SC-OGs.**

10) - consider dropping "in contrast", since there is no contrast with the previous sentence

**We thank the reviewers for their comment. We have removed “In contrast” as recommended.**

11) - consider spelling "SNAP" in the software name, also to provide clues on its mode of operation, rather than providing a definition of SNAP-OGs directly in the abstract.

**We thank the reviewers for their comment. We have clarified the etymology of OrthoSNAP in the abstract.**

12) Figure 1. Given that one of the claims the authors make in the discussion is that orthoSNAP, as opposed to other tools, can distinguish multiple paralog classes, a more complete explanation of such classes would greatly help the reader. It would then be probably more useful to provide a cartoon tailored to the classification made by orthoSNAP, rather than a general framework. This figure could then be part of the results; or combined with the current figure 1.

**We thank the reviewers for their comment. We have included an additional panel to figure 1 that also depicts what a SNAP-OG is in the context of gene M, gene N, and gene O.**

13) Last sentence of first paragraph of the introduction: The availability of more genomes actually make phylogenetic inference and thus ortholgy of genes easier rather than more complex or difficult. Could this sentence be rephrased to account for this?

**We thank the reviewers for their comment. According to results reported in Emms and Kelly, 2018 (see figure 2;** [**https://www.biorxiv.org/content/10.1101/267914v1.full.pdf**](https://www.biorxiv.org/content/10.1101/267914v1.full.pdf)**), as the number of species and the evolutionary distances among species increases, the number of SC-OGs identified using standard approaches such as OrthoFinder or reciprocal-best-directional-blast (RBBH) decreases rapidly (note, the y-axis is in log scale). Thus, we have revised the sentence to note that our ability to identify SC-OGs will become more difficult as datasets increase in size and evolutionary breadth. We also note that resolving these issues is important because increased taxon and gene sampling help improve phylogenetic inference.**

14) End of second paragraph of introduction: Rather than broad, I'd say that the past algorithms were not designed to retrieve homologs in large gene families.

**We thank the reviewers for their comment. We have decided not to revise the text because some past algorithms were designed to retrieve homologs in large gene families—for example, Agalma, which identifies maximally inclusive subtrees wherein each taxon is represented by one sequence.**

15) The final paragraph of the introduction should be part of the results, and a brief 1-3 sentences stated about the results should be what is in the introduction.

**We thank the reviewers for their comment. We have reorganized the manuscript as per their recommendation.**

16) Results, first paragraph. Do the authors have a suggested reason as to why there is variation in the results depending on lineage? It is technical or biological reason?

**We thank the reviewers for their comment. In the revised manuscript, we have discussed potential reasons that drive variation among the datasets. We focus on biological reasons, genuine duplications and deletions, especially complex evolutionary histories thereof, and technical reasons—for example, inflation parameters that are not optimized for each dataset.**

17) "Similar to taxon occupancy…." since a 50% cutoff was applied by design to both SC-OGs and SNAP-OGs, isn't this expected?

**We thank the reviewers for their comment. There is still substantial variation that may be observed in taxon occupancy. For example, SC-OGs could have a taxon occupancy of 90% whereas SNAP-OGs could be ~50%. Thus, similar taxon occupancy may not be assumed to be true.**

18) Second to last sentence of same paragraph: In terms congruence, in contradiction to this, in Budding yeast it seems that tree certainty is consistently lower in SNAP-OGs vs SG-OGs (ST6).

**We thank the reviewers for their comment. Echoing our response to comment #5, we have revised the manuscript to acknowledge SNAP-OGs have lower tree certainty than SC-OGs in the budding yeast dataset.**

19) "Resolution of this conflict": It does not look like this analysis is aimed at resolving said conflict on phylogeny, but rather to demonstrate no difference between SNAP-OGs and SC-OGs results. Please rephrase

**We thank the reviewers for their comment. We agree and have revised the manuscript accordingly.**

20) "More specifically, no differences…" this value is close to a 0.05 significance, suggesting that collapsing branches with low support values starts to highlight differences between SC-OGs and SNAP-OGs. I think these differences could be valuable to understand the nature of SNAP-OGs and deserve further analysis. Why use Benjamini-Hochberg correction?

**We thank the reviewers for their comment. We thank the reviewers for their comment. We have now included in the main text all three alternative hypotheses. All three are worth considering when evaluating gene support frequencies because they represent the three possible topologies for the three taxa in question.**

**With respect to multiple test correction, we note that examining three hypotheses will not change the number of multiple test corrections that are conducted. The reasoning is that two tests were conducted—Fischer’s exact test comparing GSF values among SC-OGs and SNAP-OGs and SC-OGs and SNAP-OGs after collapsing poorly support bipartitions. We also note that an alpha of 0.01 and 0.05 will yield the same result. That is, a p-value of 0.05 is not less than 0.05; therefore, the GSF would still not be significant. Lastly, we note that our use of a Fischer’s exact test is not guided by “test sophistication”; instead, we choose the appropriate test for the given data and experimental set up. We have also confirmed that we used the appropriate test after consulting the textbook titled “The Analysis of Biological Data” by Michael C. Whitlock and Dolph Schluter.**

**Our use of Benjamini-Hochberg multitest correction did not impact our findings. Specifically, the table below summarizes the results using seven different multi-test correction approaches. The results for multi-test correction are broadly similar across the different approaches, in part, because we are only correcting for two tests.**

| ***Multi-test correction method*** | ***GSF multi-test corrected p-value (without collapsing low supported bipartitions)*** | ***GSF multi-test corrected p-value (with collapsing low supported bipartitions)*** |
| --- | --- | --- |
| *Benjamini & Hochberg* | *0.26* | *0.05* |
| *Bonferroni* | *0.51* | *0.05* |
| *Holm* | *0.26* | *0.05* |
| *Hochberg* | *0.26* | *0.05* |
| *Hommel* | *0.26* | *0.05* |
| *Benjamini & Yekutieli* | *0.39* | *0.07* |
| *False discovery rate* | *0.26* | *0.05* |

21) Last paragraph of results: Why is this striking? One could expect that in difficult-to-resolve branches the noise would be even higher, reducing our power to detect significant differences

**We thank the reviewers for their comment. We have removed the work “strikingly.”**

22) Discussion. First 1.5 paragraphs are repetitive of the introduction. They can be deleted.

**We thank the reviewers for their comment. We have deleted these paragraphs.**

23) In certain datasets, .. This only refers to mammals. I would therefore specify

**We thank the reviewers for their comment. We specify that this phenomenon was observed in specifically the mammalian dataset.**

24) Can the authors suggest a possible interpretation of why SNAP-ORG were five times more prevalent than SC-OGs?

**We thank the reviewers for their comment. We include text in the revised manuscript that acknowledges potentially complex duplication and deletion patterns in the history of mammals as well as an ancient whole genome duplication event in budding yeasts.**

25) Qualitatively similar: Can the authors clarify what does qualitatively similar mean?

**We thank the reviewers for their comment. We clarify that both studies demonstrate the utility of expanding datasets by identifying subtrees of single-copy orthologous genes, however, we note that our pipeline also accounted for in-paralogs whereas Smith et al. did not account for in-paralogs.**

26) We also note that our algorithm: This is interesting, however it is the first time outparalogs, inparalogs, etc are mentioned (besides figure 1 legend). It does not appear to be mentioned in Methods either. More details would be useful.

**We thank the reviewers for their comment. Given that all these terms are relative to evolutionary depth and timing of duplication, we do not specify when outparalogs and inparalogs are identified by OrthoSNAP. We underscore the importance that OrthoSNAP can account for all types of paralogy whereas other software can account for some, but not all, types of paralogy.**

27) Three noteworthy differences: these differences are not in the results but in the approach, please clarify.

**We thank the reviewers for their comment and have revised the manuscript accordingly.**

28) Methods, Dataset generation: This is the first time that it is made clear that the analysis is conducted on protein sequences rather than on DNA sequences (which is implicit when talking about genes). This is an important point and should be made immediately clear, first time in the abstract.

**We thank the reviewers for their comment and have noted that these analyses were conducted with amino acid sequences in the abstract and results.**

Supplementary figures:

30) - Fig S1 Information content is minimal and fully overlaps with the text, therefore it doesn't seem a necessary figure.

**We thank the reviewers for their comment and have deleted the figure.**

31) - Fig S2 can scales be reported directly in axis ticks rather than in axis titles.

**We thank the reviewers for their comment. We have updated the figure accordingly.**

32) - Fig S7 Similar to Fig S1, the content is minimal and fully overlaps with the main text, therefore it doesn't seem a necessary figure.

**We thank the reviewers for their comment. We have removed this figure.**
