# Supplementary material for: OrthoSNAP: A tree splitting and pruning algorithm for retrieving single-copy orthologs from gene family trees
Source: PLoS Biol. 2022 Oct 13;20(10):e3001827. doi: 10.1371/journal.pbio.3001827 (PMC9595520; doi:10.1371/journal.pbio.3001827)
Supplement: Attachment — Submitted filename: response_to_reviewers_orthosnap.docx [file pbio.3001827.s020.docx]

**Editor:**

1) Thank you for your patience while we considered your revised manuscript "­­OrthoSNAP: a tree splitting and pruning algorithm for retrieving single-copy orthologs from gene family trees" for publication as a Methods and Resources at PLOS Biology. This revised version of your manuscript has been evaluated by the PLOS Biology editors, the Academic Editor, and two of the original reviewers.

Based on the reviews, we are likely to accept this manuscript for publication, provided you satisfactorily address the remaining points raised by the reviewers. Please also make sure to address the following data and other policy-related requests.

**We thank the editor for handling our manuscript. We are excited that the revised manuscript is likely to be accepted at *PLOS Biology*.**

IMPORTANT: Please attend to the following:

a) Please address the remaining minor requests from the two reviewers.

**We have addressed all reviewer minor requests in the newly revised manuscript. In addressing all comments, the clarity of our manuscript as well as the appropriate use of citations increased.**

b) Please address my Data Policy requests below; specifically, we need you to supply the numerical values underlying Figs 3ABC, 4BC, S1ABCD, S2, S3ABC, S4ABC, S5ABC, S6, S7, S8B, either as a supplementary data file or as a permanent DOI’d deposition, e.g. part of your Figshare depo (my understanding from the Figshare DOI is that this contains the raw input data, rather than the data presented in these Figure panels; please clarify).

**We have uploaded all raw data to generate the figures listed herein. Note, there is no raw underlying data for Figure S8B, thus, you will find no associated raw data.**

c) Please cite the location of the data clearly in all relevant main and supplementary Figure legends, e.g. “The data underlying this Figure can be found in S1 Data” or “The data underlying this Figure can be found in <https://figshareXXXXX>”

**We have appropriately revised the manuscript to include the location of the data for generating figures where applicable. Specifically, we included the following:**

**“The data underlying this Figure can be found in figshare (doi: 10.6084/m9.figshare.16875904).”**

**Reviewer #2:**

1) The authors have submitted a very thorough revision of their manuscript. I think it now covers what was required from my side. Just two minor comments:

**We thank you for your thorough review of our manuscript. In addressing your remaining comments, the clarity of our manuscript increased.**

2) In the sentence "30 plants, which are known to complex histories of gene duplication and loss" should be "known to have"

**The manuscript has been revised accordingly.**

3) They mention: "In comparison, 15 SC-OGs were identified in the plant dataset; 2,782 in the budding yeast dataset; and 390 in the choanoflagellate dataset", there are more SC-OGs in the yeast dataset than SNAP-OGs, could that be a typo? If not, what is the explanation for this?

**In the revised manuscript, we note one likely reason why there are more SC-OGs than SNAP-OGs. Specifically, we have included the following:**

**“Note, there are likely more SC-OGs than SNAP-OGs in budding yeasts because their genomes are relatively small and therefore do not have as many duplicate gene copies compared to other lineages, such as plants. Nonetheless, OrthoSNAP still substantially increases the number of markers in a phylogenomic data matrix.”**

**Reviewer #3:**

[identifies himself as Erich Jarvis]

1) The authors were very responsive to the reviews, and have made substantial improvements to the manuscript. This includes performing many additional analyses. All of our main concerns have been satisfied. Just two clarifications needed.

**We thank you for your review of our manuscript.**

2) The authors stated that OMA is sequence based, whereas OrthoSNAP is phylogeny based. But isn't the phylogeny of the genes based on sequence alignments?

**We apologize for the lack of clarity. In brief, OMA infers subgroups of single-copy orthologous genes using sequence similarity information alone, whereas OrthoSNAP uses phylogenetic information. To this end, we have included the following:**

**“In other words, OrthoSNAP identifies subgroups of single-copy orthologous genes by examining evolutionary histories, rather than sequence similarity values. Moreover, examination of evolutionary histories facilitates the identification of species-specific inparalogs.”**

3) The explanation of lineage sorting for not identifying all one-to-one orthologs makes sense. The authors can cite some papers where they show a high proportion of genes (5-30%) that can have incomplete lineage sorting between species (e.g. Jarvis et al 2014 Science).

**We thank the reviewer for their comment. We have now cited Jarvis et al 2014 Science as well as other manuscripts that highlight biological processes that can lead to incongruence among gene trees and the species tree.**
